# Genotyping by low-coverage whole-genome sequencing in intercross pedigrees from outbred founders: a cost-efficient approach

**DOI:** 10.1186/s12711-019-0487-1

**Published:** 2019-08-14

**Authors:** Yanjun Zan, Thibaut Payen, Mette Lillie, Christa F. Honaker, Paul B. Siegel, Örjan Carlborg

**Affiliations:** 1 Department of Medical Biochemistry and Microbiology, Uppsala University, Uppsala, Sweden; 2 Department of Animal and Poultry Sciences, Virginia Polytechnic Institute and State University, Blacksburg, VA USA

## Abstract

**Background:**

Experimental intercrosses between outbred founder populations are powerful resources for mapping loci that contribute to complex traits i.e. quantitative trait loci (QTL). Here, we present an approach and its accompanying software for high-resolution reconstruction of founder mosaic genotypes in the intercross offspring from such populations using whole-genome high-coverage sequence data on founder individuals (~ 30×) and very low-coverage sequence data on intercross individuals (< 0.5×). Sets of founder-line informative markers were selected for each full-sib family and used to infer the founder mosaic genotypes of the intercross individuals. The application of this approach and the quality of the estimated genome-wide genotypes are illustrated in a large F_2_ pedigree between two divergently selected lines of chickens.

**Results:**

We describe how we obtained whole-genome genotype data for hundreds of individuals in a cost- and time-efficient manner by using a *Tn5*-based library preparation protocol and an imputation algorithm that was optimized for this application. In total, 7.6 million markers segregated in this pedigree and, within each full-sib family, between 10.0 and 13.7% of these were fully informative, i.e. fixed for alternative alleles in the founders from the divergent lines, and were used for reconstruction of the offspring mosaic genotypes. The genotypes that were estimated based on the low-coverage sequence data were highly consistent (> 95% agreement) with those obtained using individual single nucleotide polymorphism (SNP) genotyping. The estimated resolution of the inferred recombination breakpoints was relatively high, with 50% of them being defined on regions shorter than 10 kb.

**Conclusions:**

A method and software for inferring founder mosaic genotypes in intercross offspring from low-coverage whole-genome sequencing in pedigrees from heterozygous founders are described. They provide high-quality, high-resolution genotypes in a time- and cost-efficient manner. The software is freely available at https://github.com/CarlborgGenomics/Stripes.

**Electronic supplementary material:**

The online version of this article (10.1186/s12711-019-0487-1) contains supplementary material, which is available to authorized users.

## Background

Genotyping by next-generation sequencing has emerged as a rapid, high-throughput approach to obtain high-density genotypes in large populations [1, 2]. The downside of this approach is that confident assignment of individual genotypes requires costly deep sequencing, which restricts its application for large association panels. Cost-efficient approaches, such as reduced-representation sequencing (RRS; [3]) and genotype imputation from low-coverage sequencing data [4–7], have been developed to reduce the cost of genotyping large populations. While RRS reduces the cost by sequencing only a fraction of the genome without losing confidence in individual calls, the genotype imputation approach is designed for low-coverage sequence data and subsequent genotype imputation against a set of selected high-confidence haplotypes either from external reference individuals or from haplotypes reconstructed in the low-coverage sequenced individuals. Compared to RRS, genotype imputation can provide high-confidence genome-wide genotypes at a similar cost, which makes it a compelling approach for a wide range of applications [4–8].

Several algorithms have been developed for genotype imputation based on low-coverage sequencing data. Some were developed for general pedigrees and data, while others were targeted for more specific applications, resulting in variation in their performances depending on how well underlying assumptions about the data are met. In populations with unrelated individuals, the individual genomes are often assumed to consist of short mosaics of ancestral haplotypes. Examples of software that have been developed for genotype imputation in such applications include Beagle [9], MaCH [10], and Shapeit2 [11–13]. These software perform well for their intended applications but lose considerable performance when applied to data with ultra-low coverage (< 1×) from experimental populations [14]. In such populations, relatedness of the individuals results in the segregation of longer haplotypes. By developing methods that account for these relationships, much lower sequence coverage (< 0.1×) is required to resolve, for example, the founder genome mosaics that are inherited by the offspring from the mating of two inbred parents [7, 8, 15]. Multiple methods and software exist for analyses of such ultra-low coverage sequencing data in intercrosses between pairs of inbred founders, including FSFHap [4], LB-impute [6], and TIGER [7]. This principle has also been successfully extended to populations that are founded by multiple inbred parents, with implementations in software such as Mpimpute [16] and Reconstruct [17].

There is a dearth of alternatives for analysing data from populations that were bred by mating two or more outbred (heterozygous) founders. For such populations, both genotype imputation and inference of founder mosaics in the offspring are more challenging due to the unobserved phases in the pedigree founders. A software useful for genotype imputation in such data is STITCH [5], which can perform this task by using ultra-low coverage data in both unrelated and pedigreed populations. To reconstruct founder mosaics for linkage and QTL mapping, even fewer options exist because the methods developed for inbred populations described above [6–9, 16, 18] are not immediately applicable for such populations. Recently, a method was reported to perform such reconstruction while allowing for heterozygosity of parents and offspring in both bi- and multiparental populations [14]. However, the accompanying software that implements the algorithm (magicImpute) requires a commercial platform. A recent article [19] describes an algorithm for calling, phasing, and imputation of genotypes from low-coverage sequence data in pedigreed populations using single-locus peeling, multi-locus peeling, and hybrid peeling, but the performance of the accompanying software, AlphaPeel was not evaluated here since it was published after this paper was completed.

Here, we report an approach and its associated software that facilitates cost- and time-efficient inference of founder mosaic genotypes in experimental crosses from outbred (heterozygous) founders using very low coverage (< 0.5×) sequencing data. Although our approach has several similarities to earlier methods that were developed for inbred populations from bi-parental crosses, and uses one of these in part of the implemented pipeline, it provides extensions that are essential for the efficient estimation of founder mosaics in outbred crosses with multiple segregating founders. The properties of our method were illustrated by re-genotyping an F_2_ intercross [20, 21] between the Virginia lines of broiler chickens that were divergently selected for body weight [22–24]. Our results demonstrate that this method provides high-quality genome-wide founder mosaic genotypes with crossover events that were estimated with greater resolution and at a lower cost than that of reduced representation approaches based on a few hundred selected and individually genotyped genetic markers.

## Methods

**Fig. 1:**
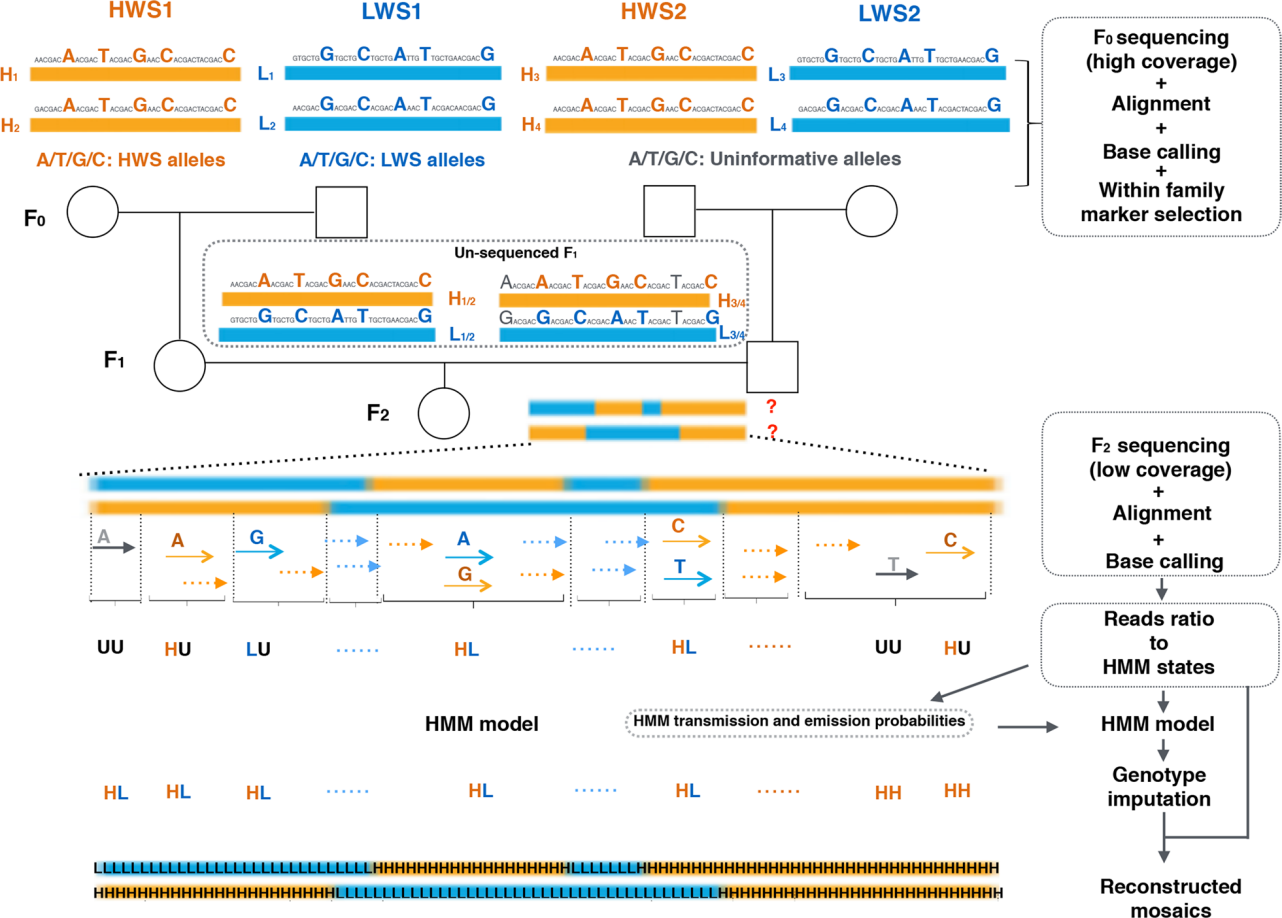
Reconstruction of the founder mosaic genotypes in an F_2_ individual from a multi-parent F_0_–F_2_ family with outbred (heterozygous) founders, using low-coverage sequence data. Informative markers (blue/yellow large font) are identified based on their fixation for alternative alleles in the pairs of deep-coverage sequenced F_0_ founders from the divergent lines (HWS1 and HWS2 vs. LWS1 and LWS2) in the family. Blue and yellow bars represent chromosomes that originate from the high (yellow) and low (blue) founder lines, respectively. Uninformative markers that segregate in at least one founder (black small font) are discarded from further analyses. The F1 individuals used as parents for the intercross (F2) offspring do not need to be sequenced, as they are heterozygous for all selected informative markers. The F_2_ individuals are sequenced to low-coverage and the reads are mapped to the selected informative markers. Then the founder mosaic genotypes (illustrated for one autosome by the blue and yellow bars) are inferred across the genome using the read mappings to the within-family informative markers using a Hidden Markov Model (HMM) developed for this task for inbred line crosses [7]

When an F_2_ intercross population is founded by intercrossing multiple outbred founders from two parental lines, the population can be divided into nuclear families with one F_2_ offspring and four F_0_ parents. The genomes of the offspring in each of these nuclear full-sib families will be mosaics of the eight haplotypes of the four F_0_ founders, as illustrated in Fig. 1. The linkage phases in the F_0_ genomes are, however, unknown, which means that the line origin of the alleles in the F_2_ offspring cannot be inferred with confidence in regions of the genome where the F_0_ founders are heterozygous (Fig. 1). Using whole-genome sequencing, markers can be detected where the divergent founders are fixed for alternative alleles (Fig. 1). Using these markers only, the founder mosaic genotypes in the entire F_2_ intercross generation can be imputed efficiently, one F_2_ full-sib nuclear family at a time. In the following sections, we describe in detail the approach that is outlined in Fig. 1. It is implemented in a freely available software pipeline that can be downloaded at https://github.com/CarlborgGenomics/Stripes.

### Inference of founder mosaic genotypes in intercross individuals from a cross between outbred founders

A more detailed, step-by-step description of the approach to estimate the founder mosaic genotypes outlined in Fig. 1 is provided below.

#### Step 1: Sequencing of F_0_ founders and F_2_ intercross individuals

Individual-based sequencing libraries were prepared for the F_0_ and F_2_ individuals. Founder individuals were sequenced to high coverage for SNP calling (15–30× recommended) and intercross individuals to low-coverage (~ 0.5× recommended).

#### Step 2: Selection of markers informative for line origin in each full-sib family

Using the high-coverage sequence data on the F_0_ individuals, markers fixed for alternative alleles in the divergent founders of each full-sib family (within-family informative markers) were identified across the genome. These markers are highlighted as large capital letters in yellow (HWS informative) and blue (LWS informative) in Fig. 1, and contrasted with the larger number of non-informative markers (small, black letters).

#### Step 3: Read calling in low-coverage sequenced intercross individuals

A computationally-efficient pipeline was implemented for read calling at the within-family informative markers in the offspring from the low-coverage sequence data (Fig. 1). First, each individual fastq file was mapped to the reference genome using “bwa mem” with default parameters. Second, the bam file was indexed and filtered using “samtools view –bh –q30 –f 0x02” to include only reads for which both pairs are uniquely mapped to the reference genome. Third, bcftools was used to obtain the number of reference and alternative allele calls using “mpileup -Oz”. Finally, a custom python script was used to reformat the obtained output to VCF format and merge the data from all individuals into a single file. This procedure was implemented in a Python snake make pipeline that is publicly available with our software release (https://github.com/CarlborgGenomics/Stripes/blob/master/scripts/fastq2vcf/Snakefile).

#### Step 4: Within-family estimation of the founder mosaic genotypes in F_2_ individuals

The founder mosaic genotypes were identified independently for each nuclear full-sib family, including four F_0_, two F_1_ and one F_2_ individuals, using the polymorphism data obtained by mapping the low-coverage reads from the F_2_ (Step 3) to the set of founder-line informative SNPs that were identified for the family (Step 2). Because all of these markers were fixed for alternative alleles in the founder lines, the dataset resembles that of an inbred population. Therefore, the founder-line mosaic genotypes of the intercross individuals in the family can be estimated using existing software developed for such populations (Fig. 1). Our pipeline uses an adapted version of the TIGER software [7] for this task. Full details regarding the model parameters and estimation procedures in TIGER are in Rowan et al. [7], which describes the algorithm and software. In short, the algorithm consists of the following steps: (i) translate the ratio of reads of the alternative alleles at each scored marker into one of six possible genotype states (HH, HU, HL, LL, LU, UU, where H represents one parental line, U represents unknown and L represents another line); (ii) estimate the ratio of reads to the two founders lines by averaging the read scores across 200 (for large chromosomes) or 50 (for small chromosomes) consecutive markers to calculate transmission and emission probabilities; and (iii) run the HMM to impute the genotypes at the markers along the chromosomes and reconstruct the founder mosaic genotypes. Here, we used chromosome-specific HMM, which differed from the original TIGER [7] algorithm in which the same HMM was used for all chromosomes of an individual. This modification was implemented to account for uneven lengths of the chromosomes in chickens and for uneven marker densities in the outbred population.

The complete analysis pipeline outlined in Steps 2 to 4 was implemented in a user-friendly pipeline manager Snakemake [25]. A detailed tutorial with instructions on installation, configuration, and launching of the analysis on demo data is available in the Github repository (https://github.com/CarlborgGenomics/Stripes). In addition, an R package was developed and released that: (i) facilitates reformatting the founder mosaic structure into a recombination mosaic matrix, where every recombination in the offspring is tagged; (ii) performs initial quality control (QC) of the data and removal of double crossover events; and (iii) exports the genotype data across bins of the desired physical length and into R/qtl format for further quality control and downstream linkage and/or QTL mapping analyses (https://github.com/CarlborgGenomics/Stripes_downstream).

### Test dataset: A large F_2_ pedigree produced from Virginia chicken lines divergently selected for body-weight

Data from a reciprocal F_2_ intercross between chickens from two divergently selected lines, obtained by bidirectional selection for body weight at 56 days of age (here referred to as the high weight selected “HWS” and low weight selected “LWS” lines) [22, 24, 26], were used to illustrate the properties of the proposed method. The base population for the lines was founded by crossing seven partially inbred lines of White Plymouth Rock chickens. The F_1_ population of the intercross was generated by mating 10 males and 17 females from the HWS to 8 males and 21 females from the LWS line. These F_0_ founders were from HWS and LWS generation 40. To generate the F_2_, 8 males and 72 females from the F_1_ were mated [20, 21]. Our analysis included 837 pedigreed F_2_ individuals with DNA available for sequence library preparation, and the 56 F_0_ founders (n_HWS_ = 27 and n_LWS_ = 29) that contributed to these F_2_ individuals.

### Whole-genome sequencing and SNP calling of pedigree founders

Libraries for high-coverage sequencing of the 56 F_0_ founders contributing to the F_2_ offspring in the pedigree were prepared using Illumina TrueSeq and sequences obtained by paired-end sequencing (2 × 150 bp) on an Illumina HiSeq X (performed by the SciLifeLab SNP&SEQ Technology platform; Uppsala, Sweden). Mapping, SNP calling, and quality control followed the Broad best practices. Reads were mapped to the chicken reference genome (galgal5; [27]) using the Burrows-Wheeler Aligner (BWA–MEM v. 0.7.13 [28]). Aligned reads were sorted and duplicate reads marked with picard (v. 2.0.1; https://broadinstitute.github.io/picard/). Base quality score recalibration (GATK 3.7) was carried out before SNP calling with HaplotypeCaller (GATK 3.7). Variants were filtered using the following criteria, a minor allele frequency (MAF) higher than 0.043, and AC and QUAL values greater than 5 and 30, respectively, which resulted in a high-quality set of SNPs for further analyses.

### Whole-genome sequencing and SNP calling of intercross individuals

A *Tn5*-based protocol [29] for low-cost and high-throughput preparation of individual sequencing libraries (~ 1€/library) was optimized for large-scale genotyping of the F_2_ intercross individuals in the pedigree. Genomic DNA was fragmented using tagmentation by *Tn5* transposase from AddGene (http://www.addgene.org/,pTXB1-Tn5; ID60240) [29]. Dual indexes were attached during PCR amplification and subsequent size selection was performed using AMPure XP beads (Beckman: A63881). The detailed procedure for library preparation, pooling and quality control is described in Zan and Carlborg [30].

Sequencing of intercross individuals was performed using an Illumina HiSeq 4000. First, the two largest F_2_ full-sib families (n = 32) were sequenced to ~ 0.8× coverage to test the quality of the prepared libraries and the implemented pooling strategy (Oklahoma Medical Research Foundation Genomics Core). The remaining F_2_ individuals (n = 805) were then sequenced to ~ 0.4× coverage by pooling ~ 200 multiplexed individuals per lane (Texas A&M Genomics and Bioinformatics Service). Demultiplexing of the dual indexed reads into individual fastq files and trimming of the adapters were done using *bcl2fastq* v2.17.1.14 (Illumina, Inc).

The low-coverage sequence data from the F_2_ individuals were mapped to the *Gallus* V5.0 reference genome [27] using the Burrows-Wheeler Aligner [28]. Only reads for which both pairs were uniquely mapped (mapping quality ≥ 30) were retained. Mpileup in Samtools 1.8 [31] was used to extract the raw information at each polymorphic site (depth and SNP read). A custom Python script was used to reformat the output such that it contained only information about the location of the polymorphism (chromosome and position), the genotype (reference and alternative allele call), and the read depth (https://github.com/CarlborgGenomics/Stripes). This procedure for calling genotypes reduced the time for computation to less than 1/10 of that of the GATK UnifiedGenotyper.

### Assessment of imputation accuracy

First, individuals with few called SNPs (genome wide average < 5 SNPs/Mb) were removed from the dataset. This was necessary because accurate genome-wide haplotype mosaic reconstruction requires a reasonably high marker density. Nearby double recombination events are biologically unlikely due to crossover interference. Cytological evidence suggests crossover interference to be absolute in regions shorter than 5 Mb on the largest chicken chromosomes [32]. The raw TIGER [7] reconstructed haplotype mosaics were filtered to remove double recombination events that were closer than 3 Mb since these are biologically most unlikely.

Sample mix-ups, DNA contaminations, and pedigree errors in the data can lead to inaccurate haplotype mosaic reconstruction and increase the number of inferred genome-wide crossover events in affected individuals. To filter out such individuals, we deleted samples for which the genome-wide genotype call rate decreased to less than 90% after removing short (< 3 Mb) double recombination events. An alternative approach would be to remove individuals that were outliers in the distribution of genome-wide recombination events that were inferred using TIGER [7]. This would lead to a very similar final set of individuals (see Additional file 1: Figure S1).

Quality of the reconstructed line origin haplotype mosaics from the low-coverage whole-genome sequence data across chromosomes 1 to 24 was evaluated by comparing them to the individual genotypes of SNPs reported previously [21]. In total, 728 of the individuals that passed our quality control filtering had genotypes at 279 of the SNPs that were reported in Wahlberg et al. [21] and that were successfully mapped from the galGal3 to the galGal5 genome assembly. The markers that were fully informative for founder-line origin were identified in each F_2_ family, ranged in number from 101 to 140 between families, and were used in this evaluation. For our approach, the genotypes—high weight homozygous (HH), heterozygous (HL), low weight homozygous (LL)—were extracted from the inferred founder mosaics at a 1-Mb resolution. Bins with TIGER imputed recombination events were excluded from the comparisons. The proportions of genotypes in agreement between the two methods per marker across individuals and across all markers per individual were used as measures of genotyping accuracy (https://github.com/CarlborgGenomics/Stripes_downstream).

## Results

Properties of the reported approach to infer founder mosaic genotypes in intercross data from outbred (heterozygous) founders were illustrated by generating and analysing data for an F_2_ population that was bred from the Virginia chicken lines divergently selected for high (HWS) and low (LWS) body weight.

### Sequencing, marker selection, read mapping and inference of founder mosaic genotypes

#### Step 1–2: Founder sequencing and identification of markers that are informative within a full-sib family

**Fig. 2:**
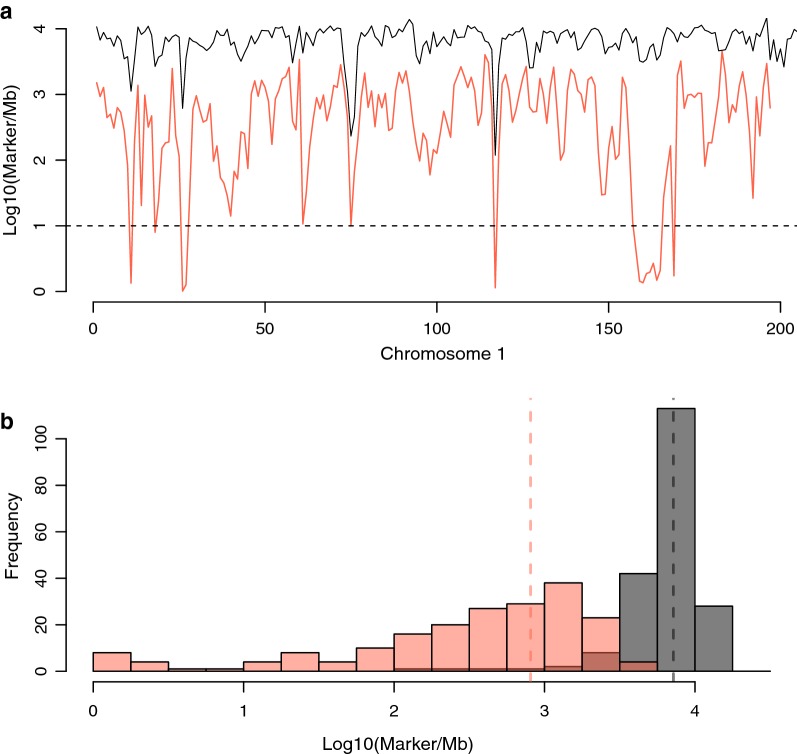
Illustrations of the density of markers fixed for alternative alleles between the HWS and LWS founders (founder line informative markers) within the F_0_–F_2_ families on chromosome 1. **a** Average number of markers in non-overlapping 1-Mb bins along chromosome 1 (y-axis; log_10_ transformed). The black and red lines represent the total number of markers that segregate in the pedigree and the average number of informative markers in the 73 families in the Virginia lines F_2_ pedigree, respectively. **b** Distribution of the average number of markers that are informative for founder line per Mb in the 73 families (x-axis; log_10_ transformed)

High coverage (~ 30×) individual sequence data detected 7,608,483 SNPs among the HWS and LWS founders (n_HWS_ = 27 and n_LWS_ = 29) that contributed to the F_2_ individuals. There were 213,946 SNPs that were fixed for alternative alleles in the sequenced individuals from the two founder lines and these were unevenly distributed across the genome (see Additional file 2: Figure S2). The numbers of SNPs that were informative within families, i.e. that were fixed for alternative alleles between the HWS and LWS founders of the individual nuclear F_0_ to F_2_ full-sib families were considerably larger, i.e. on average 840,160 SNPs per family, and were relatively evenly distributed across the genome. Thus, the average density of informative SNPs in the genome was 791 SNPs/Mb with, on average, 92% of the genomes being covered by more than 10 such SNPs per Mb in the evaluated families (Fig. 2) and (see Additional file 2: Figure S2).

#### Step 3: Low coverage sequencing of, and read mapping in, the F_2_ individuals

Sequence libraries were prepared for 837 F_2_ individuals from the intercross of the Virginia chicken lines [20, 21]. Individuals that failed in the library preparation (n = 34), or that had a low SNP coverage (< 5 SNPs/Mb; n = 14), were removed. The average sequence coverage for the remaining 789 individuals was 0.33×, which resulted in an average of 22.4% of the within-family informative SNPs (188,520, on average) having at least one mapped read within the F_0_ to F_2_ families.

#### Step 4: Genotype imputation from low-coverage sequence data in the F_2_ offspring

Whole-genome genotypes were imputed for the 789 F_2_ individuals by analysing each F_0_ to F_2_ family, in turn, with the software pipeline that we described above.

### Quality of imputed founder mosaic genotypes

**Fig. 3:**
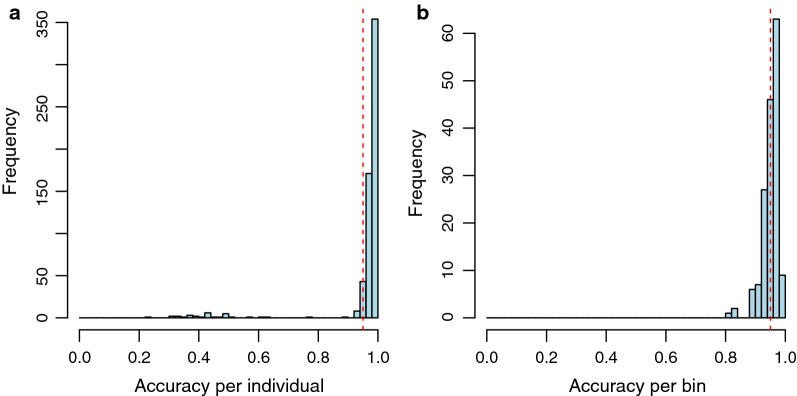
Distribution of agreements between genotypes estimated by individual SNP genotyping [20] and the founder mosaic genotypes obtained from the approach reported here across **a** all markers by individual and **b** all individuals by marker

Overall, the founder mosaic genotypes that were estimated with our method were in good agreement with the available SNP genotypes. After quality control, including removal of individuals with (i) biologically unlikely numbers of recombination events, and (ii) genotypes resulting from inferred recombination events at locations that were physically too close, the agreement between imputed genotypes using our pipeline and the previously assayed SNP genotypes in Wahlberg et al. [21] was strong. On average it reached 0.95 across the genome/individual and 0.96 per genotyped SNP across the population (Fig. 3a, b).

**Fig. 4:**
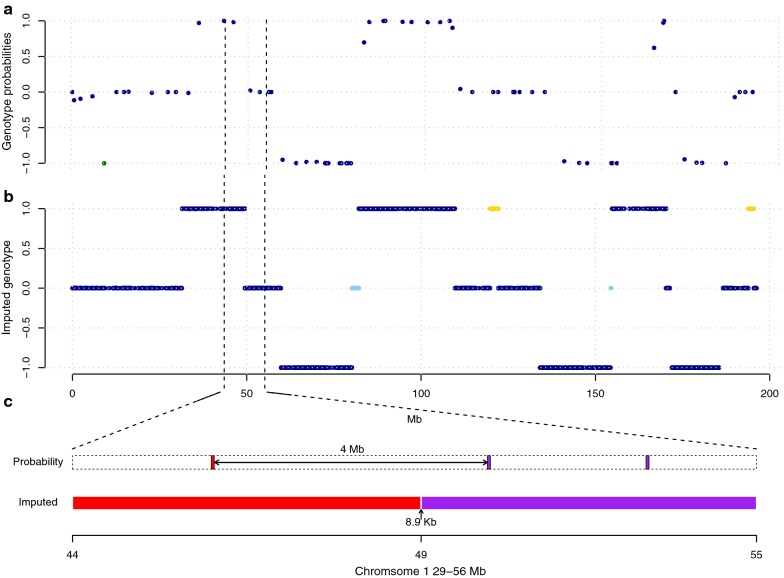
Similarity between the founder mosaic genotypes inferred from low-coverage sequence data and genotype probabilities estimated from sparse individual genotypes across chromosome 1 for one F2 chicken [20]. **a** Genotype probabilities estimated by Wahlberg et al. [20] using the algorithm described by Haley et al. [31] provided at the locations of the 74 SNPs and microsatellite markers genotyped in this pedigree. The green dot illustrates a genotype that disagrees with those imputed from sequence data. **b** Imputed genotypes from the low-coverage sequence data, where the colours of the dots indicate genotypes for which the two methods agree (dark blue), and expected heterozygous genotypes resolved (light blue) or novel putative double recombinant genotypes suggested (yellow) by imputing genotypes from the sequence data. **c** Illustration of the increased resolution from sequence-based inference of the founder mosaic for one recombination breakpoint on chromosome 1 (between 44 and 55 Mb). Genotype probabilities inferred from SNP and microsatellite data and our pipeline are illustrated as coloured bars, where red/purple indicate homozygous/heterozygous for the HWS allele, respectively

Figure 4 shows the comparison of the founder mosaic genotypes that were estimated from the low-coverage sequencing data with the method described here, with the Haley-Knott genotype probabilities [33] from Wahlberg et al. [21] for one F_2_ individual across chromosome 1. At 95.7% of the marker locations, the estimated founder mosaic genotype agreed well with the estimated genotype probabilities in Wahlberg et al. [21]. In addition, the sequence-based imputation provided much denser genome coverage (Fig. 4a vs. b). As a result, estimates of the locations of recombination breakpoints were better resolved, with 50% of them being identified on segments shorter than 10 kb (one example in Fig. 4c). The higher marker density also allowed imputation of genotypes in regions that were not covered by the set of microsatellite markers selected by Wahlberg et al. [21]. This, for example, makes it possible to resolve heterozygous regions that are flanked by homozygous regions that are agreed upon by both methods (blue in Fig. 4b). It also suggests longer putative double recombinant regions in the genome that were missed by the sparser marker set (yellow in Fig. 4b).

## Discussion

In this paper, we describe an approach and software for estimating founder mosaic genotypes in experimental crosses between outbred (heterozygous) founders from very low-coverage sequencing data. Its properties are illustrated by generating and analysing data from an F_2_ population that was produced by crossing chicken lines that were divergently selected for body weight from a common founder population.

### Comparison with another genotype imputation software (STITCH)

We compared our approach with another genotype imputation software, STITCH [5]. The imputed genotypes from STITCH [5] (run with n = 2 and K = 10), were relatively consistent with the available individual SNP genotypes (0.88/0.90 agreement for markers/individuals) and similar to those obtained with our approach before removing individuals with biologically unrealistic numbers of double-recombination events (0.90/0.89 agreement for markers/individuals). In addition to imputation accuracy, we evaluated the quality of the reconstructed founder haplotypes. As STITCH was not developed for direct estimation of founder mosaic haplotypes for QTL mapping and linkage analyses, the haplotype mosaic reconstruction was performed using custom scripts, using the single marker genotypes imputed by STITCH. Results showed that mosaics estimated from the STITCH data included an excess of recombination events [for an example (see Additional file 3: Figure S3)]. Thus, although both softwares deliver similar quality of imputed genotypes when pedigree and genotypes of founders are disregarded, when such information is available, our pipeline makes use of it to further improve genotyping quality and to estimate accurate founder mosaic genotypes in the intercross individuals. However, our software does not account for more complex crossbreeding structures that use more than two founder populations (such as MAGIC), thus making STITCH currently the best option for such datasets.

### Improved quality control by using pedigree information

Accounting for available pedigree information allows additional quality control to be performed based on the inferred number of recombination events after the founder mosaic genotypes have been estimated. First, the number of estimated crossover events across the genome should agree with its expectation given the length of the chicken linkage map [27, 34]. Second, the distances between inferred recombination events should be compatible with expectations regarding crossover interference [32] (Fig. 3). Before conducting this quality control, there was good agreement (~ 0.90) between the genotypes inferred by our approach and by STITCH using the low-coverage sequence data, and the individual SNP genotypes of Wahlberg et al. [21]. Applying filters based on these two expectations increased the level of agreement for our approach from 0.90 to 0.95 at the individual level. In addition, this allowed us to identify individuals (n = 61 or equivalent to 7%) with extremely high estimated recombination frequencies that are likely due to errors in either the recorded pedigree or mix-ups during sample preparation.

### Reconstruction of high-resolution founder mosaic haplotypes using low-coverage sequencing data

Our proposed strategy to reconstruct the founder mosaic haplotype structure using low-coverage sequencing data provides more genotyping information than classical approaches at a considerably lower cost. The founders of the pedigree were sequenced to a high coverage by using standard approaches, while F_2_ individuals were processed by a low-cost whole-genome sequencing protocol to facilitate low-coverage sequencing of hundreds of intercross individuals (< 10 €/individual, including library preparation and sequencing). This cost is lower than that of existing strategies for genotyping individuals for a few hundred SNPs, even when accounting for the cost of high-coverage sequencing of the large (n = 56) number of founder birds in our pedigree. In addition to the lower cost, there was a considerable increase in marker density, from a few hundred to several hundred thousands of imputed segregating sites across the genome.

Using pedigree information, founder mosaic genotypes can be efficiently constructed by applying approaches that were previously developed for recombinant inbred populations [7, 15]. Compared to methods that were developed for estimating founder mosaics from sparsely genotyped SNPs, such as the Haley and Knott approach [33], here, the high-density marker facilitated more precise estimation of recombination breakpoints, resulting in 50% of the recombination breakpoints being estimated to fall within 10-kb windows. Performance of the TIGER software for estimating recombination breakpoints has been thoroughly evaluated for inbred populations in [7], using simulations and experimental validation. Rowan et al. [7] reported that the individual recombination breakpoints were, in more than 90% of the cases, resolved down to 2 kb at an even lower sequence coverage than that used in our study (0.1×). Here, we found a lower resolution of the estimated breakpoints because our founders were from an outbred population, which resulted in a lower density of informative markers that were unevenly distributed across the genome. If required, it is possible to increase the resolution of recombination events by (i) also analytically tracing the makers that are fixed in two of the four F_0_ founders from one line but segregating in the two F_0_ founders from the other line (which here would increase the number of informative markers by ~ 20%), or (ii) deeper sequencing, to obtain reads at a larger proportion of the markers that are informative between the two founder lines (Fig. 2a) and (see Additional file 2: Figure S2a). However, in practice the proposed genotyping strategy is for linkage and QTL mapping of experimental crosses between outbred (heterozygous) founders. In this case, the resolution of the QTL mapping and linkage map construction is mainly limited by the actual number of recombination events in the population rather than the precision with which they are estimated, which makes marker coverage less of a concern. However, deeper sequence coverage and analytical strategies that use all types of segregating markers should be useful for future developments in which estimation of recombination events with high precision is important such as, for example, in analyses of deep intercross pedigrees, which include many more recombination events.

### Guidelines for choosing sequencing coverage

There was no obvious increase in the accuracy of the reconstructed founder mosaic genotypes after sequence coverage reached 0.05× (see Additional file 4: Figure S4). This suggests that sequencing to a lower depth than used here (0.4 ×) would have been sufficient to reach an acceptable accuracy for this population. Thus, costs could be reduced even more, without significant loss in accuracy, by lowering the sequencing coverage. On high yield sequencing platforms this would, for many species, require more unique index combinations than the 398 that are currently available for the Illumina sequencers.

Genetic divergence of the outbred founders is an important factor to be considered for determining sequencing depth. Here, the founders of the F_2_ population were from the same base population and, therefore, due to the expected low genomic divergence between these lines (Fig. 2) and (see Additional file 2: Figure S2), we opted for a high (~ 30×) coverage to identify most of the SNPs segregating in the populations. We found that 0.03% of all identified SNPs were completely fixed for alternative alleles in the two founder lines and that pairs of F_0_ individuals that were mated to generate each nuclear F_0_, F_1_ and F_2_ family were alternate homozygotes for 10.0 to 13.7% of SNPs. In populations for which the divergence between the founder populations is larger, for example in crosses between wild and domesticated populations, more divergently fixed markers are expected. Obtaining high sequence coverage for the founders is recommended, since it will benefit both the accuracy of the founder mosaics and resolution of the recombination breakpoints by revealing more of the available informative markers.

### An alternative genotyping strategy based on pooled founder sequencing

In crosses in which the founder populations are highly divergent, an alternative strategy for genotyping can be considered to reduce sequencing costs. Rather than sequencing individual founders to high coverage, libraries from the founders of each line could be pooled. The two pools, each representing one founder population, can then be sequenced to high coverage (~ 30×) to identify markers that are fixed (or nearly so) for alternate alleles in the two lines. In our F_2_ population, applying this strategy would have reduced the number of informative SNPs for genotype imputation from 840,160 to 213,946. This number of genome-wide SNPs was sufficient for reconstruction of high-quality founder mosaic genotypes. However, the uneven distribution of the informative markers along the genome would likely result in gaps for poorly covered regions (see Additional file 1: Figure S1). In this regard, our population likely represents an extreme case, because the two selected lines (F_0_s) were from a common base population founded by crossing seven partially inbred lines of White Polymouth Rock. It is worthwhile noting that while the pooled founder sequencing approach is more efficient for populations with more divergent founders, even in populations with founders from closely-related lines, it is likely still sufficient for the purpose of building high-quality linkage maps and performing genome-wide QTL analyses.

## Conclusions

In this paper, we propose and evaluate a new method for reconstruction of founder mosaic genotypes from low-coverage genome sequencing in outbred intercrosses. We applied this method to an outbred chicken F_2_ cross to illustrate how it provides high-quality, high-resolution genotypes in a time- and cost-efficient manner.

## Additional files

**Additional file 1: Figure S1.** Visualization of the number of crossovers in all F_2_ individuals. (a) Histogram of number of imputed crossover events for the 803 genotyped F_2_ individuals; b) Number of imputed crossover events in each individual, sorted by the 73 full-sib families. Individuals with low call rate (call Rate < 0.9) are coloured into red.

**Additional file 2: Figure S2.** Distribution of the between founder line informative SNPs for the evaluated F_0_-F_2_ families on chromosome 1. (a) Line graph illustrating the number of SNPs in non-overlapping 1-Mb bins across chromosomes 1 to 24 (y-axis; log_10_ transformed). The black/tomato lines represent the total number of SNPs segregating in the pedigree/the average number of informative SNPs for the 64 families in the Virgina chicken line F_2_ pedigree. (b) Histogram illustrating the average number of informative SNPs in the 73 full-sib families in the pedigree (x-axis; log_10_ transformed).

**Additional file 3: Figure S3.** Comparison of the founder mosaic in one F2 offspring obtained by using individual SNP-genotypes (a), to that obtained from our method (b) and STITCH (c) using the same low-coverage sequence data.

**Additional file 4: Figure S4.** Illustration of relationship between sequencing coverage, SNP density and imputation accuracy. (a/b) Histograms of the sequencing coverage/SNP densities for the 803 genotyped F_2_ individuals; (c/d) Scatter plots of individual coverage/SNP density vs imputation accuracy measured as proportion of sites that has same genotype with the averaged genotype probabilities estimated by Wahlberg et al. [21] using genotypes of 434 SNPs and microsatellite markers with the Haley and Knott algorithm [32].

## Data Availability

The data used to evaluate the pipeline are available on request from the corresponding author.
