# Supplementary material for: Genotyping by low-coverage whole-genome sequencing in intercross pedigrees from outbred founders: a cost-efficient approach
Source: Genet Sel Evol. 2019 Aug 14;51:44. doi: 10.1186/s12711-019-0487-1 (PMC6694510; doi:10.1186/s12711-019-0487-1)
Supplement: Supplementary file 1 — Additional file 1: Figure S1. Visualization of the number of crossovers in all F2 individuals. (a) Histogram of number of imputed crossover events for the 803 genotyped F2 individuals; b) Number of imputed crossover events in each individual, sorted by the 73 full-sib families. Individuals with low call rate (call Rate < 0.9) are coloured into red. [file 12711_2019_487_MOESM1_ESM.docx]

**Additional information for:**
Genotyping by low-coverage whole-genome sequencing in intercross pedigrees from outbred founders: a cost efficient approach

Yanjun Zan, Thibaut Payen, Mette Lillie, Christa F. Honaker, Paul B. Siegel and Örjan Carlborg

**
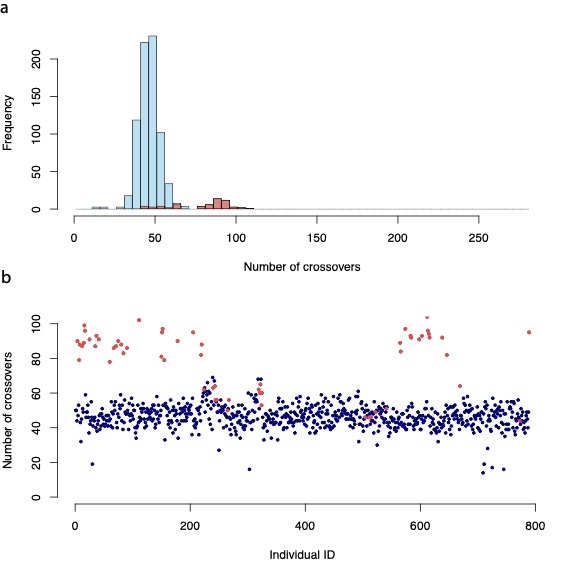
**

***Figure S1. Visualization of the number of crossover in all F_2_ individuals****. A) Histogram of number of imputed crossover events for the 803 genotyped F_2_ individuals; B) Number of imputed crossover events in each individual, sorted by the 73 full-sib families. Individuals with low call rate (call Rate <0.9) are coloured into red.*
