# Supplementary material for: Genotyping by low-coverage whole-genome sequencing in intercross pedigrees from outbred founders: a cost-efficient approach
Source: Genet Sel Evol. 2019 Aug 14;51:44. doi: 10.1186/s12711-019-0487-1 (PMC6694510; doi:10.1186/s12711-019-0487-1)
Supplement: Supplementary file 2 — Additional file 2: Figure S2. Distribution of the between founder line informative SNPs for the evaluated F0-F2 families on chromosome 1. (a) Line graph illustrating the number of SNPs in non-overlapping 1-Mb bins across chromosomes 1 to 24 (y-axis; log10 transformed). The black/tomato lines represent the total number of SNPs segregating in the pedigree/the average number of informative SNPs for the 64 families in the Virgina chicken line F2 pedigree. (b) Histogram illustrating the average number of informative SNPs in the 73 full-sib families in the pedigree (x-axis; log10 transformed). [file 12711_2019_487_MOESM2_ESM.docx]

**Additional information for:**
Genotyping by low-coverage whole-genome sequencing in intercross pedigrees from outbred founders: a cost efficient approach

Yanjun Zan, Thibaut Payen, Mette Lillie, Christa F. Honaker, Paul B. Siegel and Örjan Carlborg


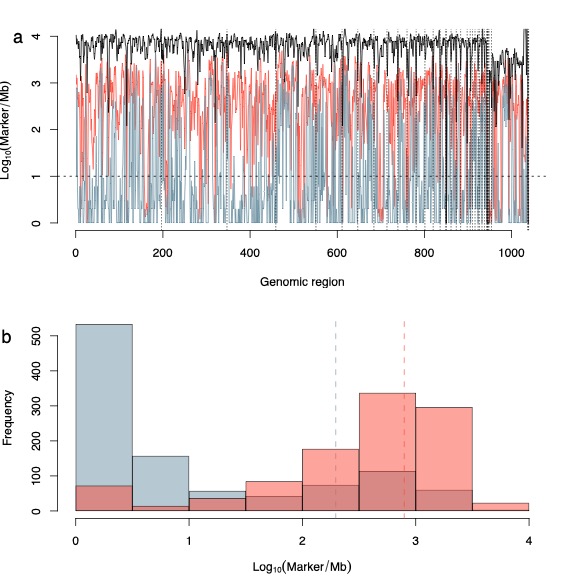


***Figure S2****. Distribution of the between founder line informative SNP markers for the evaluated F_0_-F_2_ families on chromosome 1.* ***A)*** *Line graph illustrating the number of markers in non-overlapping 1Mb bin across chromosomes 1-24 (y-axis; log_10_ transformed). The black/tomato lines represent the total number of markers segregating in the pedigree/the average number of informative markers in the 64 families in the Virgina chicken line F_2_ pedigree.* ***B)*** *Histogram illustrating the average number of informative markers in the 73 full-sib families in the pedigree (x-axis; log_10_ transformed).*
