# Supplementary material for: Genotyping by low-coverage whole-genome sequencing in intercross pedigrees from outbred founders: a cost-efficient approach
Source: Genet Sel Evol. 2019 Aug 14;51:44. doi: 10.1186/s12711-019-0487-1 (PMC6694510; doi:10.1186/s12711-019-0487-1)
Supplement: Supplementary file 3 — Additional file 3: Figure S3. Comparison of the founder mosaic in one F2 offspring obtained by using individual SNP-genotypes (a), to that obtained from our method (b) and STITCH (c) using the same low-coverage sequence data. [file 12711_2019_487_MOESM3_ESM.docx]

**Additional information for:**
Genotyping by low-coverage whole-genome sequencing in intercross pedigrees from outbred founders: a cost efficient approach

Yanjun Zan, Thibaut Payen, Mette Lillie, Christa F. Honaker, Paul B. Siegel and Örjan Carlborg


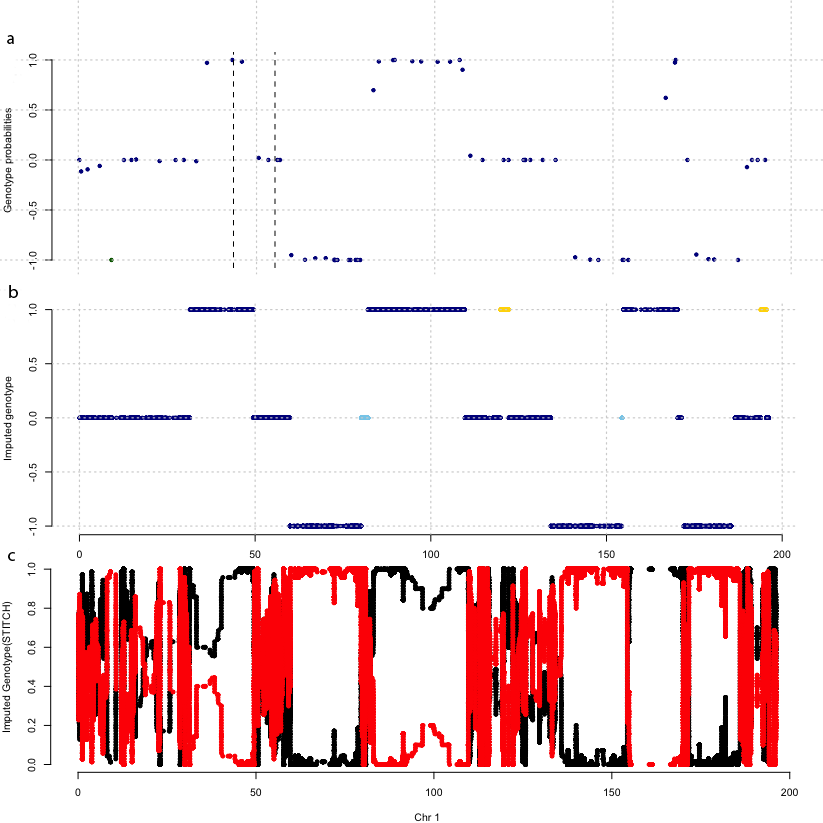


***Figure S3****. Comparison of the founder mosaic in one F2 offspring obtained using individual SNP-genotypes (A), to that obtained from our method (B) and STITCH (C) using the same low-coverage sequence data.*
