# Supplementary material for: Genotyping by low-coverage whole-genome sequencing in intercross pedigrees from outbred founders: a cost-efficient approach
Source: Genet Sel Evol. 2019 Aug 14;51:44. doi: 10.1186/s12711-019-0487-1 (PMC6694510; doi:10.1186/s12711-019-0487-1)
Supplement: Supplementary file 4 — Additional file 4: Figure S4. Illustration of relationship between sequencing coverage, SNP density and imputation accuracy. (a/b) Histograms of the sequencing coverage/SNP densities for the 803 genotyped F2 individuals; (c/d) Scatter plots of individual coverage/SNP density vs imputation accuracy measured as proportion of sites that has same genotype with the averaged genotype probabilities estimated by Wahlberg et al. [21] using genotypes of 434 SNPs and microsatellite markers with the Haley and Knott algorithm [32]. [file 12711_2019_487_MOESM4_ESM.docx]

**Additional information for:**
Genotyping by low-coverage whole-genome sequencing in intercross pedigrees from outbred founders: a cost efficient approach

Yanjun Zan, Thibaut Payen, Mette Lillie, Christa F. Honaker, Paul B. Siegel and Örjan Carlborg


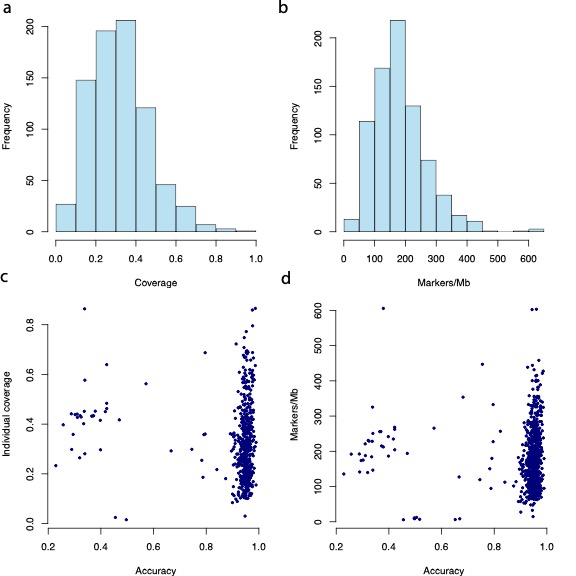


***Figure S4. Ilustration of relationship between sequencing coverage, marker density and imputation accuracy.*** ***A/B)*** *Histograms of the sequencing coverage/marker densities for the 803 genotyped F_2_ individuals;* ***C/D)*** *Scatter plots of individual coverage/marker density vs imputation accuracy measured as proportion of sites that has same genotype with the averaged genotype probabilities estimated by* (Wahlberg et al., 2009) *using genotypes of 434 SNP and microsatellite markers with the Haley and Knott algorithm* (Haley et al., 1994)*.*
